# A Pharmacological Comparison of Two Isomeric Nicotinic Receptor Agonists: The Marine Toxin Isoanatabine and the Tobacco Alkaloid Anatabine

**DOI:** 10.3390/md18020106

**Published:** 2020-02-11

**Authors:** Hong Xing, Sunil Keshwah, Anne Rouchaud, William R. Kem

**Affiliations:** Department of Pharmacology and Therapeutics, College of Medicine, University of Florida, Gainesville, FL 32610, USASunil.Keshwah@uchospitals.edu (S.K.);

**Keywords:** acetylcholine, alkaloid, Alzheimer’s disease, anabaseine, anabasine, anatabine, inflammation, isoanatabine, nemertine, nicotine, nicotinic acetylcholine receptor, tobacco, toxin

## Abstract

Many organisms possess “secondary” compounds to avoid consumption or to immobilize prey. While the most abundant or active compounds are initially investigated, more extensive analyses reveal other “minor” compounds with distinctive properties that may also be of biomedical and pharmaceutical significance. Here, we present an initial in vitro investigation of the actions of two isomeric tetrahydropyridyl ring-containing anabasine analogs: isoanatabine, an alkaloid isolated from a marine worm, and anatabine, a relatively abundant minor alkaloid in commercial tobacco plants. Both compounds have a double bond that is distal to the piperidine ring nitrogen of anabasine. Racemic isoanatabine and anatabine were synthesized and their S- and R-enantiomers were isolated by chiral high pressure liquid chromatography (HPLC). Both isoanatabines displayed higher efficacies at α4β2 nicotinic acetylcholine receptors (nAChRs) relative to the anatabines; R-isoanatabine was most potent. Radioligand binding experiments revealed similar α4β2 nAChR binding affinities for the isoanatabines, but R-anatabine affinity was twice that of S-anatabine. While the two anatabines and S-isoanatabine were highly efficacious agonists at α7 nAChRs, R-isoanatabine was only a weak partial agonist. The four compounds share an ability to stimulate both α4β2 and α7 nAChRs, a property that may be useful in developing more efficacious drugs to treat neurodegenerative and other medical disorders.

## 1. Introduction

Due to its many effects on the human organism, (S)-nicotine, the most abundant pharmacologically active constituent of the commercial tobacco plant *Nicotiana tabacum*, is one of the most thoroughly investigated natural products. It is also one of the most addictive of drugs and, when administered by smoking, is the major cause of preventable human death. In recent decades, much has been learned about the myriad central nervous system nicotinic acetylcholine receptors (nAChRs) that are activated by nicotine and similar “nicotinoid” compounds [1,2,3,4,5]. It has been demonstrated that nicotine and some other nAChR agonists have pro-cognitive, anxiolytic, analgesic, and anti-inflammatory properties that might be useful in the treatment of certain neurodegenerative, neurodevelopmental, inflammatory, and drug addiction disorders [4,5,6]. Several drug candidates targeting nAChRs have undergone clinical tests for some of these potential therapeutic indications, but only one, varenicline, has reached the clinic [7,8,9,10].

The mammalian organism contains more than fifteen different nAChRs, which are expressed in neurons, skeletal muscles, glia, macrophages, keratinocytes, lung epithelia, and immune cells [1]. Each nAChR is composed of five homologous, protein subunits, which form a barrel-like membrane protein complex. When this membrane protein is naturally activated by the neurotransmitter ACh, endogenous choline, or nicotine, its ion channel opens, causing a membrane potential change that may be sufficient to transiently generate a nerve or muscle impulse in an electrically excitable cell or elevate its intracellular calcium concentration. The nAChRs are conveniently classified according to their subunit compositions. Some are homo-oligomeric, containing five copies of a single subunit. Most receptors containing the α7 subunit are homo-oligomeric and their ion channel displays selectivity for calcium ions, which activate intracellular signaling cascades [11]. Hetero-oligomeric nAChRs are more common and include several brain, autonomic nervous system, and neuromuscular nAChRs. The α4β2 receptors are the major heteromeric receptor subtype in the brain and spinal cord. Nicotine has an exceptionally high affinity for β2-containing nAChRs, which mediate many of its physiological and behavioral effects, including tobacco addiction. Recently, other nAChRs have been found to participate in certain brain circuits involved in nicotine and other drug addictions [12].

Although nAChRs have been extensively investigated during the past three decades, once they were cloned and expressed in cell lines, much remains to be learned about their in vivo functions and their potential as therapeutic targets. In the 1980s, it was reported by many laboratories that the membrane concentrations of some nAChRs, especially the α4β2 subtypes, are greatly reduced in cognitive circuits of the brain during the progression of neurodegenerative diseases (Alzheimer’s and Parkinson’s) and are not expressed normally in some other central nervous system disorders [13]. Thus, initial pharmaceutical interest targeting α4β2 or α7 nAChRs focused on behavioral effects of nicotinoids, including enhancement of cognition and the treatment of tobacco addiction. Efforts to design and develop drugs that can selectively stimulate or antagonize these central nervous system receptors are still in progress. In addition, recent investigations demonstrating the involvement of nAChRs in acute (sepsis, pancreatitis, etc.) and chronic (rheumatoid arthritis, multiple sclerosis, etc.) inflammatory diseases have opened a new therapeutic frontier that ultimately may lead to better treatments of these medical conditions [14,15].

**Figure 1:**
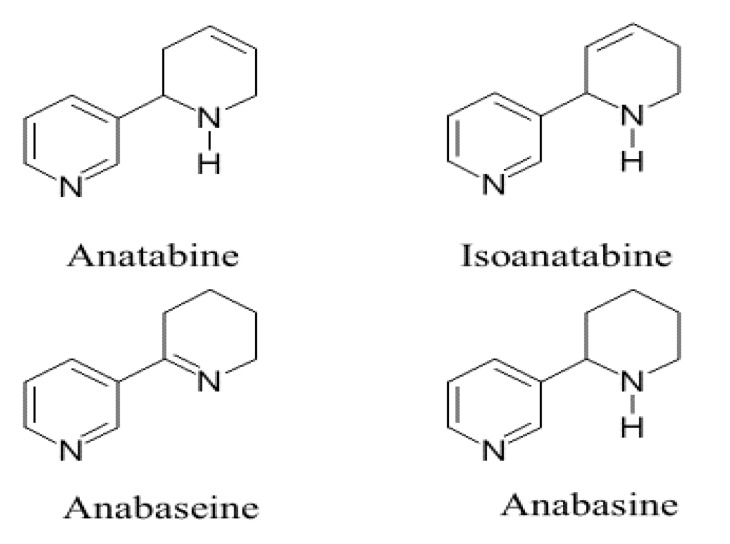
Structures of the two alkaloids, tobacco anatabine and nemertine isoanatabine, compared with anabasine and another tetrahydropyridyl ring isomer, anabaseine. Anatabine and anabasine are known to largely occur as (S)-enantiomers; the possible chirality of natural isoanatabine is not yet known. Both anabaseine and anabasine have been found in marine (nemertine) worms and in ants.

Nicotine, anabasine, and anabaseine (Figure 1) are potent but unselective agonists at most nAChRs, which limits their therapeutic use. However, many drug candidates, including analogs of nicotine and anabaseine, have been reported to selectively affect a single nAChR subtype, which reduces the possibility of adverse effects. In spite of numerous compounds being developed and the recent appearance of selective allosteric modulators, nAChR-targeted drug design is still an immature field of investigation. It has benefited greatly from the availability of a large number of potent natural products (“molecular probes”) that act upon nAChRs. These include natural agonists such as anatoxin [16], anabaseine [17,18], and epibatidine [19], as well as competitive antagonists such as *Erythrina* [20] and *Delphinium* alkaloids [21]. 

This paper will describe in vitro studies of two “minor” nicotinoid natural products: (1) isoanatabine, which we isolated from a nemertine (nemertean) worm, *Amphiporus angulatus*, and (2) anatabine, the most abundant minor alkaloid of the commercial tobacco plant (Figure 1). Both compounds are isomers differing only in the position of a single double bond in an otherwise saturated piperidine ring in the “parent” compound anabasine, which is present in wild tobacco [22]. The pharmacological properties of anabasine [23,24] and the related anabaseine [25,26] have been investigated, but not as extensively as those of nicotine. 

The hoplonemertine *A. angulatus* contains many pyridine alkaloids. We initially isolated two major compounds, 2,3′-bipyridyl and a tetrapyridyl we named nemertelline [27,28]. The worm inhabits the northwestern Atlantic, North American Arctic, and both northern Pacific coasts. It lives under rocks in the lower intertidal and sublittoral zones and preys upon amphipod crustaceans. Its potential predators include crabs and blenny fishes that live under the same rocks. While 2,3′-bipyridyl rapidly produces convulsions and paralysis of crustaceans, nemertelline causes a less rapid, sleep-like paralysis in crustaceans. Many (>10) “minor” compounds, including isoanatabine, have been isolated from *A. angulatus* by high-pressure liquid chromatography and identified by nuclear magnetic resonance and mass spectrometric methods (Kem et al., in preparation). The presence of 3-methyl-2,3′-bipyridyl and isoanatabine has already been reported [29,30,31]. 

Over a century ago, anatabine was isolated from tobacco leaves, and its structure reported by Spath’s laboratory [32]. It is the most abundant minor alkaloid in tobacco, representing about 4% of the alkaloids present in tobacco leaves; nicotine represents >90% of the alkaloids present [33]. Due to it is relative resistance to metabolism, it has become a useful biomarker of tobacco use [34]. Several anatabine syntheses and in vivo pharmacological studies have been published, but its in vitro effects on nAChRs have not been published. For that reason, we investigated the effects of anatabine and isoanatabine on the two major central nervous system (CNS) nAChRs.

## 2. Results

### 2.1. Experiments with Human nAChRs Expressed in Xenopus Oocytes

**Figure 2:**
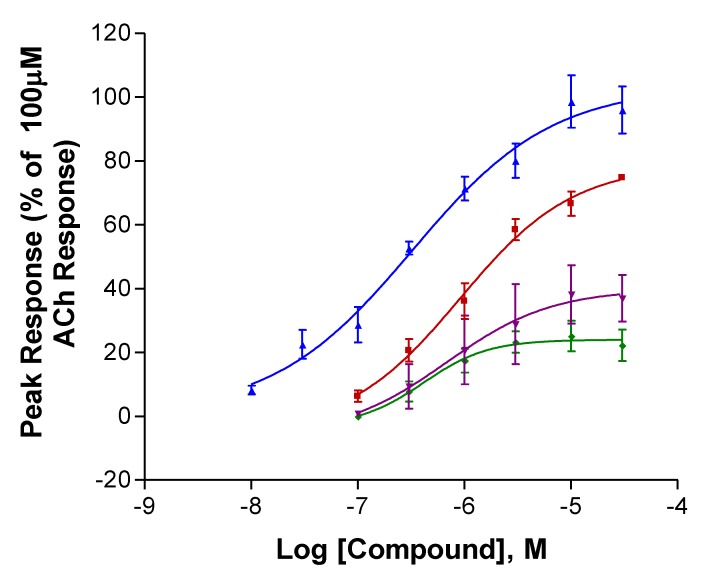
Activation of human α4β2 neuronal nicotinic acetylcholine (nACh) receptors expressed in *Xenopus* oocytes. All peak responses were normalized with respect to the response of the cells to 100 μM ACh. R-Isoanatabine (Blue, ▲), S-Isoanatabine (Red, ■), R-Anatabine (Green, ⧫), S-Anatabine (Purple, ▼). SEM bars are included.

Complete concentration–response curves for the stimulation of human α4β2 nAChRs are shown in Figure 2. Table 1 provides efficacy (current response, I_max_) and potency (EC_50_, the concentration producing half the maximal response for the compound) estimates from the curves in Figure 2; these estimates are arranged to facilitate comparisons between different enantiomers of the same compound (top half) and between similar enantiomers of the different compounds (bottom half). The two isoanatabines displayed significantly greater efficacies than the two anatabines (Table 1). R-isoanatabine’s potency was greater than that of S-isoanatabine (Student’s t-test P = 0.020). The efficacy of S-anatabine was larger than that of R-anatabine (P = 0.029). Comparing the S-enantiomers of the two compounds, S-isoanatabine’s efficacy was significantly greater than that of S-anatabine (P = 0.0061). Comparing the R-enantiomers of the two compounds, R-isoanatabine’s efficacy was also greater than that of S-anatabine (P < 0.001). 

The two isoanatabines were more efficacious and the two anatabines were less efficacious at human α4β2 nAChRs compared with S-anabasine (I_max_ = 78%, Kem et al., in preparation). However, all compounds except R-isoanatabine still had efficacies at human α4β2 nAChRs that were inferior to that of nicotine (Xing et al., submitted). All four compounds displayed potencies at human α4β2 nAChRs that were greater than that for S-anabasine (EC_50_ = 4 µM, Kem et al., in preparation). 

**Figure 3:**
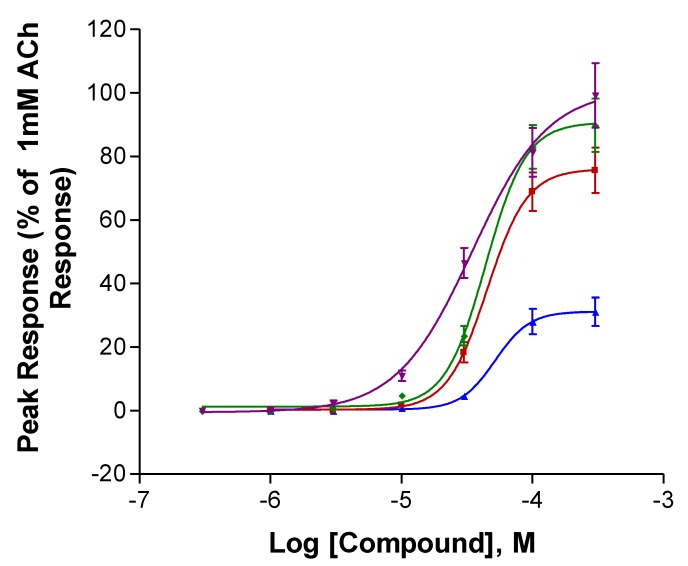
Activation of human α7 (right) neuronal nACh receptors expressed in *Xenopus* oocytes. R-Isoanatabine (Blue, ▲), S-Isoanatabine (Red, ■), R-Anatabine (Green, ⧫), S-Anatabine (Purple, ▼). All peak responses were normalized with respect to the response of the cells to 1 mM (α7) ACh. SEM values are included.

The two anatabines displayed the highest efficacies at α7 receptors (Figure 3 and Table 2). However, the α7 receptor potencies of all four compounds were rather similar, the EC_50_s ranging from 52 to 70 µM. All four compounds possessed potencies that were similar to that of nicotine (EC_50_ = 65 µM, Xing et al., submitted). 

### 2.2. Radioligand Binding Experiments with Rat Brain nAChRs

We utilized [^3^H]-cytisine displacement to measure the relative affinities of the isoanatabines and anatabines for rat brain high-nicotine-affinity receptors, which have been shown to be >90% of the α4β2 subtype. At the low [^3^H]-cytisine concentration (0.3 nM) employed in these experiments, one can be confident that binding to the high-sensitivity α4_2_β2_3_ stoichiometry receptors is being measured, since the low-sensitivity α4β2 receptors have a much lower affinity (~50X) for cytisine [35]. 

**Figure 4:**
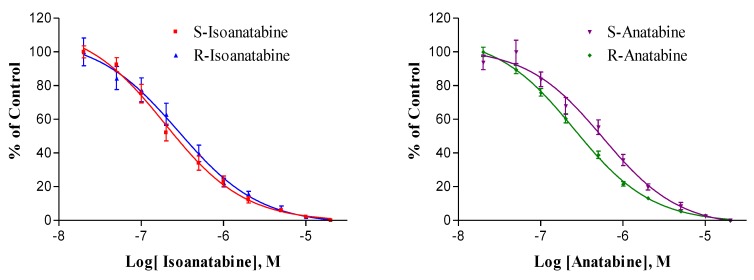
Competition binding assay of (S and R)-Isoanatabines (left) and (S and R)-Anatabines (right). Displacement of [^3^H]-cytisine from rat brain membranes. R-Isoanatabine (Blue, ▲), S-Isoanatabine (Red, ■), R-Anatabine (Green, ⧫), S-Anatabine (Purple, ▼). Each point represents the mean of 12 replicates (three experiments, identical concentrations tested in quadruplicate); SEM bars are shown.

Compound affinities for α4β2 nAChRs, measured by radioligand binding methods, are shown in Figure 4, and the binding constants are presented and compared in Table 3. The affinities of the four compounds were rather similar, being in the 100–250 nM range. The binding affinity of S-isoanatabine was slightly higher than that of the S-anatabine (P = 0.005). Although the affinities of the two isoanatabines were not statistically different, the R-anatabine K_i_ was half of the S-anatabine K_i_ (P = 0.001). By comparison, the nicotine K_i_ for rat brain receptors is approximately 2 nM (Xing et al., submitted). The affinities measured by displacement of cytisine binding were approximately 100× higher than would be predicted from the EC_50_ values of the compounds. This is not surprising since other binding studies using cytisine and related radioligands (nicotine and epibatidine) have found much higher affinities than would be inferred from functional estimates. Radioligand binding experiments measure ligand interaction under steady-state conditions, unlike functional experiments and, thus, are greatly affected by high affinity for the desensitized state of the α4β2 receptors. Thus, one cannot expect a strict proportionality between EC_50_ and K_i_ estimates.

Our binding data for rat brain α7 receptors were limited to single [^125^I]-α-bungarotoxin displacement experiments (ten compound concentrations, each with four identical samples) for S-anatabine (K_i_ = 236 ± 20 nM) and R-anatabine (K_i_ = 680 ± 120 nM).

## 3. Discussion

Insertion of the double bond in an otherwise piperidine ring flattens this part of the ring, preventing the 3,4 (isoanatabine) or 4,5 position (anatabine) carbons from taking a half-chair or half-boat conformation. Our main observations are that the presence of a double bond in either position increases agonist potency at α4β2 nAChRs but decreases potency at α7 nAChRs, relative to S-anabasine. Anabasine displays about a 10-fold lower potency than nicotine for α4β2 nAChRs, but an approximately threefold higher potency at α7 nAChRs (Xing et al., in preparation). Anabasine binds about fivefold less tightly to rat α4β2 nAChRs than to α7 nAChRs [25]. The larger ring size of anabasine seems less favorable for binding and activation of α4β2 nAChRs [35]. Addition of the double bond appears to partially compensate for the enlargement of the ring. Crystal structures of nicotine bound to *Lymnaea* acetylcholine binding protein (AChBP, Protein Data Bank IUW6 [36]) and anabaseine bound to *Aplysia* AChBP (Protein Data Bank 2WNL [37]) indicate that these nicotinoid agonists occupy most of the volume of the binding site. Introduction of the 3,4-double bond in isoanatabine enhances efficacy at α4β2 receptors relative to anabasine, but 4,5-double bond introduction in anatabine reduces its efficacy at these receptors relative to anabasine. Perhaps this is due to the proximity of the 3,4-double bond to the 2-position carbon, to which the pyridyl ring is connected; the lone sp2 hydrogen at the 3-position may improve the relative orientations of the two rings for isoanatabine binding at the α4β2 binding site. Crystal structures of anabasine, isoanatabine, and anatabine bound to nAChRs or to AChBPs could provide a firmer basis for interpreting the pharmacological effects of an added double bond in the two isomeric compounds.

The S- and R-enantiomers for each compound did not display large differences in their interactions with the two nAChRs, as was also observed for the anabasine enantiomers [23, and (Kem et al., in preparation)]. An exception was the much lower efficacy of R-isoanatabine at α7 nAChRs (Figure 3 and Table 2) relative to S-isoanatabine. S-anatabine accounts for approximately 85% of tobacco anatabine [33]. Anabasine also occurs predominantly as the S-form, as does nicotine [33].

Biologically, the presence of a distal double bond in isoanatabines and anatabines provides the organism (nemertine and tobacco plant) with a toxin capable of interacting more potently and efficaciously with α4β2 nAChRs than either anabasine or anabaseine [25]. Thus, the organism probably acquires an enhanced ability to neutralize predators and prey (nemertine) or herbivores (tobacco) by targeting an additional subtype of nAChR more effectively than anabasine or anabaseine. 

Until now, the in vitro agonist properties of anatabine have not been investigated. However, anatabine has been shown to have anti-inflammatory actions, both in vivo and in vitro [38,39]. Racemic anatabine was marketed as an herbal supplement called Anatabloc by Rock Creek Pharmaceuticals. Several studies supported by this company have shown that anatabine, in addition to its anti-inflammatory actions, has procognitive [40,41,42,43], neuro-protective [44], immune disease modulating [45,46], and smoking cessation actions. Anatabine partially substitutes for nicotine in drug discrimination behavioral assays but does not have the addictive potency of nicotine [47,48]. Racemic anatabine released dopamine from striatal slices obtained from adult mice [49] but failed to reduce the threshold for intracranial electrical stimulation, a useful measure of its reinforcing ability [50]. A recent investigation also found that anatabine has anti-depressant effects in mice [51]. Most (if not all) of these studies apparently used synthesized, racemic anatabine. Ours is the first study where both enantiomers were tested separately.

While similar in vivo studies have yet to be carried out with an isoanatabine, the similarity of its in vitro properties with those of anatabine suggest that isoanatabine will also have similar in vivo actions. Both isomeric analogs of anabasine—isoanatabine and anatabine—have therapeutic potential and merit further investigation. It was recently reported that agonists that co-stimulate both of the major brain nAChRs have greater efficacy in protecting brain cells from β-amyloid [52]. Similarly, the precognitive effects of varenicline seem to depend on stimulation of both α4β2 and α7 nAChRs [53]. These interesting findings will stimulate further investigations of agonists that have this co-stimulatory ability at a common concentration. Isoanatabine and anatabine are compounds which should be capable of co-stimulating these two receptors, particularly since it has been shown that α7 nAChRs are maximally stimulated at concentrations that are as much as 10 × lower than are found to be effective when measured by peak current measurements such as the ones we used in this study. It will also be important to evaluate the effects of isoanatabine and anatabine on autonomic ganglion and neuromuscular nAChRs, which are potential sites for their adverse effects. 

## 4. Materials and Methods

### 4.1. Isoanatabine and Anatabine Syntheses

Isoanatabine synthesis was achieved via a procedure using simple and inexpensive starting materials [31]. The key step of our synthesis was introduction of the pyridyl group via a Bruylants reaction, reacting 1-methyl-2-cyano-3-piperideine [54] with 3-pyridyl magnesium chloride. N-Demethylation of the N-oxide of the N-Me isoanatabine by FeSO_4_ in MeOH at 10 °C (non-classical Polonovski reaction) led to isoanatabine. The ^1^H and ^13^C NMR spectra of racemic isoanatabine were identical to those of natural isoanatabine (Kem et al., in preparation]. Racemic anatabine analytical data were also in agreement with published data. Enantio-separation was achieved by chiral-phase HPLC (Chiralcel OJ-H column, 1 cm × 25 cm, Daicel Chemical Industries Ltd.; Affiliate: Chiral Technologies, Inc., West Chester, PA, USA). For anatabine, an initial 10 min development with hexane-2.5% ethanol-0.1% trifluoroacetic acid-0.1% diethylamine was followed by a 40 min linear gradient attaining hexane-10% ethanol-0.1% TFA-0.1% DEA after 40 min. The S-anatabine peak occurred at 43 min and the R-anatabine peak at 47 min. For isoanatabine, an initial 10 min development with 98% (v/v) hexane-2.0% ethanol-0.15% trifluoroacetic acid-0.1% diethylamine was followed by a linear gradient attaining 80% hexane-10% ethanol-0.1% TFA-0.1% DEA after 40 min. The S-isoanatabine peak occurred at 33 min and the R-anatabine peak at 40 min. See Tang et al. [55] for more details regarding chiral separations of nicotine alkaloids with this column.

### 4.2. Two-Electrode Voltage-Clamp Analysis of Human Brain nAChRs

Because the two nAChRs of interest often occur on the cell membranes of the same neurons, we chose to separately investigate the functional effects of the two alkaloids using the frog (*Xenopus*) oocyte expression system, which has been widely used to investigate the agonistic or antagonistic effects of many compounds acting on nAChRs. It has been especially useful for investigating α7 nAChRs, due to the difficulties that were initially encountered in functional expression of this particular homomeric nAChR in mammalian cells. We measured the peak current response of an oocyte to a given concentration of the compound and expressed its amplitude with respect to the mean response to ACh before and after the compound test concentration. These EC_50_ values are minimal estimates of α7 receptor potency, as it has been shown that the peak current is not a direct measure of receptor activation by the concentration being tested, since the rate of desensitization of this receptor exceeds the rate of equilibration of the compound with the receptors on the oocyte surface [56]. The actual EC_50_ values of the compounds may be as much as 10-fold lower than our peak current estimates due to this difference in the method of estimating the α7 receptor responses [56]. However, since we were interested in comparing the relative efficacies and potencies of the four isomeric compounds and their rates of diffusion to the receptors are expected to be almost identical, our peak current measurements should allow quantitative comparisons. 

Oocytes were harvested from *Xenopus laevis* frogs obtained from NASCO Scientific Co. (Ft. Atkinson, WI, USA) according to an accepted Kem laboratory Institutional Animal Care Committee (IACUC) approved protocol. We have already described the methods of oocyte injection, perfusion, rapid solution changes, and recording in considerable detail [57]. Oocytes were injected with identical amounts (usually 12 ng) of human α4 and β2 mRNAs for α4β2 nAChR expression, or 20 ng of human α7 mRNAs for α7 nAChR expression. The two-electrode voltage-clamp method was used for recording peak responses (holding Em-50 mV for α4β2 and-60 mV for α7 receptors). For convenience, we only measured peak current responses to various concentrations of the two compounds and normalized the data relative to ACh responses before and after the test pulse; the standard ACh concentrations were 100 µM for α4β2 nAChRs and 1000 µM for α7 nAChRs, since the latter receptor is less sensitive to ACh (Xing et al., submitted). The oocyte was no longer used if the current response to ACh decreased more than 20% during the experiment. Compounds dissolved in Barth’s saline (pH 7.3) were rapidly administered by an AutoMate perfusion system. Compound applications were separated by a 5 min wash period. We utilized GraphPad Prism software to analyze the data, always assuming a single homogeneous receptor population.

### 4.3. Radioligand Binding

[^3^H]-Cytisine was purchased from New England Nuclear and iodinated α-bungarotoxin [^125^I]-BTX from Amersham. Unstripped whole male Sprague-Dawley rat brains obtained from Pel-Freez Biologicals (Rogers, AZ, USA) were prepared in Tris binding saline (pH = 7.4) containing 2 mg/mL BSA. [^3^H]-Cytisine binding experiments were performed according to [57] with several modifications [58]. Rat brain membranes were incubated with [^3^H]-cytisine at 4 °C for 4 h. Then, 200 μg of membrane protein was incubated in a final volume of 500 μL binding saline at 4 °C for 4 h, while binding assays with ^125^I-bungarotoxin were incubated at 37 °C for 3 h to assure that equilibrium was reached. Nonspecific binding was measured in the presence of 1 mM (S)-nicotine hydrogen tartrate salt. Reactions were terminated by vacuum filtrations through Whatman GF/C filters (presoaked in PEI) using a Brandel cell harvester. Bound [^3^H]-cytisine and ^125^I-bungarotoxin were measured with liquid scintillation and gamma counters, respectively. Data were analyzed using GraphPad Prism software (San Diego, CA, USA). The data were fit by nonlinear regression analyses to a sigmoidal dose-response with variable slope. K_i_ values were calculated using the Cheng-Prusoff equation (K _i_ = IC_50_/(1 + [RL]/K_d RL_)) with a K_d RL_ value for each radioligand that had been previously determined. IC_50_ is the median inhibition concentration of the experimental compound and [RL] is the concentration of the radioligand.

## 5. Conclusions

Both isoanatabine enantiomers were more potent α4β2 agonists than the two anatabines; R-isoanatabine was the most efficacious and potent agonist. Both anatabine enantiomers and S-isoanatabine were strong partial agonists at the human α7 receptor relative to R-isoanatabine. Further pharmacological analysis of these two isomeric piperideine analogs of anabasine may provide structural explanations for the effects of the added double bond and differences between isoanatabines and anatabines. The ability of these isomeric natural products to co-stimulate the two major brain nAChRs may provide a means of increasing therapeutic efficacy beyond what would be achieved by solely stimulating one of these nAChRs.

## Figures and Tables

**Table 1:** Comparison of *Xenopus* oocyte-expressed human α4β2 nAChR efficacy (I_max_) and potency (EC_50_) estimates for the four compounds. These efficacy and potency estimates were obtained by Prism analysis of the data used to construct the curves in Figure 2 n = number of oocytes tested. Table 1, Table 2 and Table 3 are organized to facilitate comparison of the two enantiomers (above) of each isomer and the same enantiomers of the two isomers (below). SEM values are included.

Compound	I_Max_ (%ACh)	P	EC_50_ (µM)	P	N
S-isoanatabine	78.7 ± 1.9	0.02 *	1.01 ± 0.33	0.12	4
R-isoanatabine	102 ± 5.7		0.31 ± 0.09		4
S-anatabine	43.2 ± 4.3	0.029 *	2.65 ± 1.4	0.3	3
R-anatabine	25.0 ± 5.2		0.74 ± 0.21		8
S-isoanatabine	78.7 ± 1.9	0.0061 ^ŧŧ^	1.01 ± 0.33	0.36	4
S-anatabine	43.2 ± 4.3		2.65 ± 1.4		3
R-isoanatabine	102 ± 5.7	<0.001 ^ŧŧŧ^	0.31 ± 0.09	0.099	4
R-anatabine	25.0 ± 5.2		0.74 ± 0.21		8

* P value from *t*-test between S- and R-enantiomers of the same compound (S-/R-Isoanatabine and S-/R-Anatabine): * < 0.05. ^ŧ^ Value from *t*-test comparing the same (S- or R-) enantiomers of different compounds: ^ŧŧ^ *p* < 0.01, ^ŧŧŧ^ *p* < 0.001.

**Table 2:** Comparison of *Xenopus* oocyte human α7 nAChR efficacy (I_max_) and potency (EC_50_) estimates for the four compounds. These efficacy and potency estimates were obtained by Prism analysis of the data used to construct the curves in Figure 2. n = numbers of oocytes tested. SEM values are included.

Compound	I_max_ (% ACh)	P	EC_50_ (µM)	P	n
S-Isoanatabine	81.9 ± 9.1	0.0007 ***	52.3 ± 13	0.61	9
R-Isoanatabine	35.4 ± 5.3		61.3 ± 12		9
S-Anatabine	113 ± 20	0.788	69.7 ± 30	0.569	9
R-Anatabine	105 ± 16		51.8 ± 6.5		8
S-Isoanatabine	81.9 ± 9.1	0.193	52.3 ± 13	0.599	9
S-Anatabine	113 ± 20		69.7 ± 30		9
R-Isoanatabine	35.4 ± 5.3	0.003 ^ŧŧ^	61.3 ± 12	0.50	9
R-Anatabine	105 ± 16		51.8 ± 6.5		8

* P value from t-test for S- and R-enantiomers of the same compound (S-/R-Isoanatabine and S-/R-Anatabine): *** *p* < 0.001. ^ŧ^ Value from t-test comparing the same (S- or R-) enantiomers of different compounds: ^ŧŧ^ *p* < 0.01.

**Table 3:** Isoanatabine and anatabine binding to rat brain α4β2 nAChRs measured by displacement of [^3^H]-cytisine-specific binding. There were no statistically significant differences (P > 0.05) between isoanatabine and anatabine or for S-anatabine versus R-anatabine. SEM values are shown. By comparison, S- and R-anabasine K_i_ values for rat brain α4β2 nAChRs measured under similar conditions were 1100 and 910 nM, respectively [24]. n = total number of replicates.

Compound	K_i_ (nM)	P	Hill Slope	P	n
S-Isoanatabine	108 ± 14	0.13	1.08 ± 0.15	0.40	12
R-Isoanatabine	136 ± 11		0.94 ± 0.05		12
S-Anatabine	249 ± 32	0.001 **	0.98 ± 0.09	0.70	12
R-Anatabine	119 ± 16		0.94 ± 0.06		12
S-Isoanatabine	108 ± 14	0.005 **	1.08 ± 0.15	0.59	12
S-Anatabine	249 ± 32		0.98 ± 0.09		12
R-Isoanatabine	136 ± 11	0.39	0.94 ± 0.05	0.99	12
R-Anatabine	119 ± 16		0.94 ± 0.06		12

* P value from *t*-test for S- and R-enantiomers of the same compound (S-/R-Isoanatabine and S-/R-Anatabine): ** < 0.01. ^ŧŧ^ Value from *t*-test comparing the same (S- or R-) enantiomers of different compounds: ^ŧŧ^ *p* < 0.01.
